# Effects of salinity on photosynthetic traits, ion homeostasis and nitrogen metabolism in wild and cultivated soybean

**DOI:** 10.7717/peerj.8191

**Published:** 2019-12-09

**Authors:** Abd Ullah, Mingxia Li, Javaria Noor, Akash Tariq, Yuan Liu, Lianxuan Shi

**Affiliations:** 1 Institute of Grassland Science, Northeast Normal University, Key Laboratory of Vegetation Ecology, Ministry of Education, Changchun, China; 2 Department of Botany, Islamia College University, Peshawar, Khyber Pakhtunkhwa, Pakistan; 3 State Key Laboratory of Desert and Oasis Ecology, Xinjiang Institute of Ecology and Geography, Chinese Academy of Sciences, Urumqi, China; 4 Cele National Station of Observation and Research for Desert-Grassland Ecosystems, Cele, Xinjiang, China; 5 Key Laboratory of Biogeography and Bioresource in Arid Zone, Chinese Academy of Sciences, Urumqi, China; 6 University of Chinese Academy of Sciences, Beijing, China

**Keywords:** Nitrogen metabolism, Ions, Photosynthesis, Glycine Soja, Glycine max

## Abstract

**Background:**

Carbon and nitrogen metabolism need to be highly regulated to achieve cell acclimation to changing environmental conditions. The understanding of physio-biochemical responses of crops to salinity stress could help to stabilize their performance and yield. In this study we have analyzed the roles of photosynthesis, ion physiology and nitrate assimilation toward saline/alkaline stress acclimation in wild and cultivated soybean seedlings.

**Methods:**

Growth and photosynthetic parameters, ion concentrations and the activity of enzymes involved in nitrogen assimilation were determined in seedlings of one wild and one cultivated soybean accession subjected to saline or alkaline stresses.

**Results:**

Both saline and alkaline stresses had a negative impact on the growth and metabolism of both wild and cultivated soybean.The growth, photosynthesis, and gas exchange parameters showed a significant decrease in response to increasing salt concentration. Additionally, a significant increase in root Na^+^ and Cl^–^ concentration was observed. However, photosynthetic performance and ion regulation were higher in wild than in cultivated soybean under saline and alkaline stresses. Nitrate reductase (NR) and the glutamine synthetase/glutamate synthase (GS/GOGAT) cycle showed a significant decrease in leaves of both genotypes. The reduction in the GS/GOGAT cycle was accompanied by high aminating glutamate dehydrogenase (NADH-glutamate dehydrogenase) activity, indicating the assimilation of high levels of NH_4_^+^. A significant increase in the activities of aminating and deaminating enzymes, including glutamate dehydrogenase (GDH), alanine aminotransferase (AlaAT) and aspartate aminotransferase (AspAT), was observed, probably due to the high glutamate demand and maintenance of the Krebs cycle to correct the C: N status.

**Conclusions:**

Cultivated soybean was much more stress sensitive than was the wild soybean. The decrease in growth, photosynthesis, ion regulation and nitrogen assimilation enzymes was greater in cultivated soybean than in wild soybean. The impact of alkaline stress was more pronounced than that of saline stress. Wild soybean regulated the physiological mechanisms of photosynthesis and nitrate assimilation more effectively than did cultivated soybean. The present findings provide a theoretical basis with which to screen and utilize wild and cultivated soybean germplasm for breeding new stress-tolerant soybean.

## Introduction

Soybean (*Glycine max*) is an important economic crop, supplying 30% of the world’s edible oil and 69% of its edible protein (Zhang et al., 2016). However, cultivated soybean is generally salt-sensitive and needs genetic improvement to make better use of alkaline and salinized soils (Wang & Li, 2011). Wild soybean (*Glycine soja*) is the ancestor of cultivated soybean (Duanmu et al., 2015). In comparison with cultivated species, wild soybean relatives have greater genetic diversity and greater resistance and tolerance to various biotic and abiotic stresses, respectively (Xue et al., 2014). Therefore, studies to compare the stress response in both wild and cultivated accessions are of the utmost importance, to improve abiotic stress tolerance and hence the adaptability of cultivated soybean genotypes.

Soil salinity is a global environmental problem that can severely affect plant and crop growth (Munns, 2002). The problem of salinity is increasing rapidly and it has been estimated that more than 50% of the cultivated land will be salinized by the year 2050 (Wang, Vinocur & Altman, 2003). Neutral salt stress is caused by neutral salts (such as NaCl and Na_2_SO_4_), where as alkaline salt stress is caused by alkaline salts (including NaHCO_3_ and Na_2_CO_3_) (Shi & Sheng, 2005). Together, both salt stresses affect ∼932 million hectares of land worldwide (Rao et al., 2008). Soil salinity usually causes osmotic changes, cellular ion imbalance, photosynthetic inhibition, reduced nutrient acquisition, membrane disorganization, oxidative stress, metabolic toxicity, and membrane leakage (Munns & Tester, 2008).

In this context, plant-based solutions are needed and the analysis of traits related to salt tolerance is essential, to develop crops which survive in salt-affected soils (Ashrafi et al., 2014). Usually, plants cope with salt stress by synthesizing certain organic compatible solutes such as betaine, soluble sugars, and amino acids. These compatible solutes possess osmoprotectant properties which can help plants to survive extreme osmotic stress (Negrao, Schmockel & Tester, 2017). Therefore, studying photosynthesis and nitrate assimilation under salt stress assumes critical importance in plant stress physiology. Photosynthesis is a vital physiological process that supplies fixed carbon and chemical energy. Glucose and sucrose accumulation is an adaptive response to salt stress and plays a significant role in the normalization of plant metabolism by facilitating protein turnover and compatible solute production (Singh et al., 2015).

Salt stress usually affects the ribulose biphosphate carboxylase-oxygenase activity, photosystem II light-capturing efficiency and electron transport ability (Sivakumar, Sharmila & Saradhi, 2000; Yang et al., 2006), as well as photosynthetic gas exchange parameters in various crop plants (James et al., 2002; Lawlor & Tezara, 2009). The harmful effects of salinity are mainly associated with toxic accumulations of Na^+^ and Cl^−^ concentration which can inhibit the process of photosynthesis (Teakle & Tyerman, 2010). Na^+^ accumulation appears to be more toxic for *G. soja* (Luo, Yu & Liu, 2005), whereas Cl^−^ accumulation is more toxic for *G. max* (Chen & Yu, 2007). Salt ions also interfere with plant nitrogen assimilation (Debouba et al., 2006; Queiroz, Sodek & Haddad, 2012), by inhibiting NO_3_^−^ reduction and NH_4_^+^ assimilation, and affecting the activities of nitrate reductase (NR), (Debouba et al., 2006; Bybordi & Ebrahimian, 2011; Gao et al., 2013; Shao et al., 2015; Meng et al., 2016), glutamine synthetase (GS) and glutamate synthase (GOGAT) enzymes (Kwinta & Cal, 2004; Debouba et al., 2006; Wang et al., 2012; Shao et al., 2015; Meng et al., 2016; Liu & Von Wirén, 2017). In contrast, salt stress usually stimulates both the aminating and deaminating reactions of the glutamate dehydrogenase (GDH) enzyme. Deaminating glutamate dehydrogenase (NADH-GDH) can incorporate NH_4_^+^ into glutamate and plays a crucial role in ammonium detoxification (Wang et al., 2014). Aminating glutamate dehydrogeanse (NAD-GDH) triggers the oxidation of glutamate to 2-oxoglutarate and delivers carbon skeletons under stress conditions (Liu & Von Wirén, 2017). Glutamine and glutamate are precursors for the biosynthesis of other amino acids and further nitrogenous compounds (Forde & Lea, 2007; Kusano et al., 2011). Aspertate aminotransferase (AspAT) and alanine aminotransferase (AlaAT) catalyze the reversible transfer of an amino group between glutamate and oxaloacetate/pyruvate, leading to the formation of 2-oxoglutarate and aspartate/alanine, respectively (Forde & Lea, 2007). Salt ions usually induce the activities of both the AspAT and AlaAT enzymes (Surabhi et al., 2008; Gao et al., 2013). The reversible reactions of aminotransferases convert ketoacids to amino acids and maintain the correct carbon to nitrogen ratio. It is important for plants to maintain a correct C: N ratio for adjustment and adaptation to various abiotic stresses, including salt stress (Coruzzi & Zhou, 2001). However, the adaptability of coupled carbon and nitrogen metabolism to saline/alkaline stress in soybean seedlings is unknown. Therefore, the aim of this study was (a) to analyze and compare the changes in growth, photosynthetic ability, ion accumulation and nitrogen metabolism in wild and cultivated soybeans under different saline and alkaline stresses, (b) to unravel the physiological stress tolerance mechanisms, and (c) to provide a scientific basis for the breeding of new salt-tolerant soybean varieties.

## Materials & Methods

### Plant materials and salt treatments

The experiment was conducted outdoors and the pots were sheltered from rain, at Northeast Normal University Changchun, Jilin, Northeast China (43°05′∼45°15′N, 124°18′∼127°05′E).The mean ± standard error (SE) temperature was 18.5 ± 1.5 °C at night and 26 ± 2 °C in the daytime, and the humidity was 60% ±5%. The seeds of the wild soybean genotype (W; Huinan 06116) and the cultivated soybean accession (C; Jinong 24) were provided by Jilin Province Crop Breeding Center of New Varieties. The seeds were sown in plastic pots (15 cm in diameter) with a hole in the bottom ( two cm in diameter) and filled with 2.5 kg of thoroughly washed sand. Initially, the seedlings were watered with full-strength Hoagland solution every morning. At the third compound leaf stage, thirty-five pots of uniform seedlings were selected and split into seven sets (per accession) for stress treatment: three sets of five pots each for saline stress (NaCl and Na_2_SO_4_, at a 1:1 molar ratio), three sets of five pots each for alkaline stress three (Na_2_CO_3_ and NaHCO_3_, at a 1:1 molar ratio) and one set of five pots for the positive control (untreated). Both saline and alkaline stresses consisted of three concentrations (10, 20 and 30 mmol L^−1^, *n* = 5). All solutions were prepared in 1 ×Hoagland solution. The stress was gradually implemented with 10 mmol L^−1^ of saline or alkaline stress solution for the first two days to allow the seedlings to adapt to salt stress, followed by the respective treatments, i.e., 10, 20 or 30 mmol L^−1^ for two weeks. The leaves of the two soybean accessions were then harvested from three randomly selected pots per treatment and immediately frozen in liquid nitrogen and stored at −80 °C for further analysis (see below).

### Growth parameters

After the soybean seedlings were harvested, shoot height, root length, shoot and root fresh weight, and shoot and root dry weight were measured (Shao et al., 2016).

### Gas exchange parameters

After two weeks of stress treatment, gas exchange parameters of fully expanded leaves were measured at photosynthetically active radiation (PAR) of 1,200 ± 50 µmolm^−2^ s^−1^, using a portable open flow gas exchange system LI-6400 (LI-COR, USA) at 11:00 am. The concentration of CO_2_ in the atmosphere was 380 ± 5 cm^3^ m^−1^. Air humidity and temperature were about 50% and 24 ° C, respectively. The results for net CO_2_ assimilation rate (*P*_*n*_), transpiration rate (*E)*, stomatal conductance (*G*_*s*_) and ratio of sub-stomatal to atmospheric CO_2_ concentration (*C*_*i*_*/C*_*a*_) were expressed in units of µmol CO_2_m^−2^ s^−1^, µmol H_2_O m^−2^ s^−1^, mol m^−2^ s^−1^, and cm^3^m^−3^, respectively. The water-use efficiency parameter (*WUE*) was calculated as the ratio of *P*_*n*_*/E*. Measurements were carried out for each treatment from each of three replicate pots and the mean values were recorded.

### Measurement of photosynthetic pigments

Dry leaf samples (0.1 g) were immersed in 80% acetone/anhydrous ethanol mixture (1:1) to extract the photosynthetic pigments completely. Photosynthetic pigments were determined using a spectrophotometer (Type UV-754; Shanghai Accurate Scientific Instrument Co., China) to measure absorbance at 440, 645 and 663 nm.

Photosynthetic pigment concentrations (mg g^−1^) were calculated using the following equations:

\begin{eqnarray*}\text{Chlorophyll a (Chl a)}& =9.784{A}_{663}-0.990{A}_{645} \end{eqnarray*}

\begin{eqnarray*}\text{Chlorophyll b (Chl b)}& =21.426{A}_{645}-4.650{A}_{663} \end{eqnarray*}

\begin{eqnarray*}\text{Total chlorophyll (Chl t)}& =\mathrm{Chla}+\mathrm{Chlb}=5.134{A}_{663}-20.436{A}_{645} \end{eqnarray*}

\begin{eqnarray*}\text{Carotenoids (Car)}& =4.695{A}_{440}-0.268Chlt \end{eqnarray*}

\begin{eqnarray*}\text{Content}({\mathrm{mgg}}^{-1})& =\mathrm{concentration}\times \mathrm{volume}(10 \mathrm{mL})/\mathrm{sample~ dry~ weight}\nonumber\\\displaystyle (50\times 1{0}^{-3}\mathrm{g}) \end{eqnarray*}

(Holm, 1954).

### Mineral ion concentration determination

Each dry root samples (0.05 g) was treated with deionized water (four mL) at 100 °C for 40 min and then centrifuged at 3000 × g for 15 min.The supernatant was collected and the extraction was repeated twice, with the extracts combined and adjusted to 15 mL. This combined supernatant was used for the determination of NO_3_^−^, Cl^−^, SO_4_^2−^, H_2_PO^4−^ and oxalate (C_2_O_4_^2−^) anions, using ion chromatography (DX-300 ion chromatographic system, AS4A-SC chromatographic column, CDM-II electrical conductivity detector, mobile phase: Na_2_CO_3_/NaHCO_3_ = 1.7/1.8 mM; Dionex, Sunnyvale, CA, USA). An atomic absorption spectrophotometer (Super 990F, Beijing Purkinje General Instrument Co. Ltd. Beijing, China) was used to determine the concentrations of Na^+^, K^+^, Ca^2+^, Mg^2+^, Fe^2+^, B^3+^, Cu^2+^, Mn^2+^, Zn^2+^, and P^3+^ cations (Jiao et al., 2018)

### Enzyme extract preparation

Fresh leaf samples were ground in a chilled mortar and homogenized with 50 mM Tris-HCl buffer (pH 7.5), containing 500 mM EDTA, 1 mM MgCl_2_, 10 mM β-mercaptoethanol, and 0.5% polyvinylpyrrolidone (PVP). The homogenate was centrifuged at 12,000 × g for 10 min at 4 °C and the supernatant was retained to assay *in vitro* activities of nitrate reductase (NR), glutamine synthetase (GS), glutamine synthase (GOGAT), glutamate dehydrogenase (GDH), alanine aminotransferase (AlaAT) and aspartate aminotransferase (AspAT), as well as protein concentration.

### Nitrate reductase  (NR) activity

NR activity was determined by the sulfamate colorimetric method (Cruz & Martins Loução, 2002). The reaction mixture, containing 200 mM KNO_3_, 5 mM EDTA, and 0.15 mM NADH in 100 mM phosphate buffer (pH 7.5) was incubated for 1 h at 30 °C. Then two mL of sulfanilamide and two mL of α-naphthylamine reagents were added and the absorbance was read at 540 nm. The enzyme activity was expressed in units of µmol g^−1^ protein.

### Glutamine synthetase (GS) activity

GS activity was assayed according to the method of Oaks et al. (1980). The reaction mixture, which included 100 mM Tris–HCl buffer (pH 7.4), 80 mM MgSO_4_, 20 mM sodium glutamate, 20 mM cysteine, 2 mM EGTA, ATP solution (0.7 mL) and enzyme extract, was incubated for 30 min at 37 °C . Then, one mL of ferric chloride reagent (0.37 M FeCl_3_ and 0.2 M trichloroacetic acid in 0.5 M HCl) was added and centrifuged at 5,000 × g for 10 min at 4 °C. The absorbance was read at 540 nm and enzyme activity was expressed in units of µmol g^−1^ protein.

### Glutamine synthase (GOGAT) activity

GOGAT activity was assayed according to the method of Rachim & Nicholas (1985). The reaction mixture contained 20 mM L-glutamine, 100 mM α-ketoglutaric acid, 10 mMKCl, and 3 mM NADH in 25 mM Tris–HCl (pH 7.6). The reaction was started by adding the enzyme extract. The NADH oxidation was monitored by continuously recording the absorbance at 340 nm. The oxidation of 1 µmol NADH per min was considered to be an enzyme unit (µmol g^−1^ protein).

### Glutamate dehydrogenase (GDH) activities

Aminating glutamate dehydrogenase (NADH-GDH) activity was evaluated according to the method of Groat & Vance (1981). For deaminating glutamate dehydrogenase (NAD-GDH) activity, the reaction mixture consisted of 23.1 mM *α*-ketoglutaric acid, 231 mM NH4Cl, 30 mM CaCl2, and 6 mM NADH in 100 mM Tris–HCl buffer (pH 8.0), and the reaction was started by adding the enzyme extract. The reaction mixture for NAD-GDH consisted of 100 mM L-glutamic acid, and 1 mM NAD in 100 mM Tris–HCl buffer (pH 8.8), plus the enzyme extract. The NADH oxidation (NADH-GDH) or NAD reduction (NAD-GDH) activity was measured spectrophotometrically at 340 nm. The enzyme activity was expressed in units of µmol g-1 protein.

### Aminotransferase activities

The alanine aminotransferase (AlaAT) and aspartate aminotransferase activities were measured spectrophotometrically at 340 nm in the alanine-pyruvate/aspartate-oxaloacetate directions coupled with oxidation of NADH by lactate dehydrogenase and malate dehydrogenase, respectively (De Sousa & Sodek, 2003). For AlaAT assay, the enzyme extract was mixed with a buffer solution of 500 M L-alanine, 15 mM *α*-oxoglutarate, 0.15 mM NADH and 5 units of lactate dehydrogenase in 100 mM Tris–HCl buffer (pH 7.5) (De Sousa & Sodek, 2003). The reaction mixture for AspAT consisted of 200 M L-aspartate, 5 mM EDTA, 12 mM 2-oxoglutarate, 0.15 mM NADH, and five units of malate dehydrogenase in 100 mM Tris–HCl buffer (pH 7.2), to which was added the enzyme extract (De Sousa & Sodek, 2003). The aminotransferases were measured using the absorption coefficient for NADH (ε = 6.22 mM^−1^ cm^−1^). They were expressed in units expressed in µmol g^−1^protein.

### Protein determination

Protein concentration was measured following the method of Bradford (1976), using bovine serum albumin as the standard.

### Data analysis

All measurements were repeated three times, and the data organized in Microsoft Excel 2007. The data values are presented as mean ± standard error (SE). The data were analyzed statistically by two-way analysis of variance (ANOVA) in SPSS (v.13.0;IBM, Armonk, NY, USA) and significant differences among treatment means were detected at *P* < 0.05 by pairwise multiple comparisons, using Duncan’s multiple range test. Sigma Plot 10.0. was used to draw the figure graphics, all of which show data points and error bars as the mean ± SE. To aid interpretation, principal component analysis (PCA) of the ionome variation analysis, photosynthetic parameters and nitrogen assimilation enzyme activities were performed for both species.

## Results

### Changes in plant growth performance

The growth performances of the seedlings of the wild and cultivated soybean accessions exhibited obvious differences in response to stress treatment (Tables 1 and 2). In response to increasing concentrations of either salt treatment, the shoot and root fresh and dry weights of both soybean accessions all decreased significantly. The two stresses significantly reduced shoot and root length in both the wild and cultivated accessions (Tables 1 and 2; *P* < 0.05). Growth reduction was greater in the cultivated soybean accession than in the wild soybean accession, and the negative impact of alkaline stress was more obvious than that of saline stress, i.e 12.67% decrease (in 30 mmol L^−1^ saline stress) and 18.17% decrease (in 30 mmol L^−1^ alkaline stress) in the wild accession, compared with 34.96% (in 30 mmol L^−1^ saline stress) and 34.96% (in 30 mmol L^−1^ alkaline stress) in the cultivated soybean accession. Root length in the wild soybean accession increased significantly by 1.99% under 10 and 20 mmol L^−1^ saline stress, relative to the unstressed control (Tables 1 and 2).

**Table 1:** Changes in shoot height, shoot fresh and dry weight of wild sand cultivated soybeans under control and salt stress conditions. The values are shown as mean ± SE. Different letters (A–G and a–g) mark significantly different values at *P* < 0.05 according to Duncan’s method. W, wild soybean; C, cultivated soybean; CK, Control; L-NS, M-NS, and H-NS represent low (10 mmol L ), medium(20 mmol L^−1^) and high (30 mmol L^−1^) concentrations of saline salt stress, respectively; while L-AS, M-AS and H-AS represent low (10 mmol L^−1^), medium (20 mmol L^−1^) and high (30 mmol L^−1^) concentrations of alkaline salt stress, respectively.

Variety	Treatment	Shoot height (cm)	Shoot fresh weight (g)	Shoot dry weight (g)
	CK	90.80 ± 2.69**A**	10.67 ± 1.18**A**	2.60 ± 0.40**A**
	L-NS	86.20 ±S 2.14**AB**	9.73 ± 0.24**A**	2.37 ± 0.11**A**
	M-NS	83.22 ± 3.50**AB**	8.77 ± 1.05**A**	2.32 ± 0.06**A**
W soybean	H-NS	79.30 ± 0.70**BC**	8.53 ± 0.32**A**	2.23 ± 0.18**A**
	L-AS	82.52 ± 4.22**ABC**	9.13 ± 0.33**A**	2.00 ± 0.17**AB**
	M-AS	77.63 ± 2.46**BC**	8.00 ± 0.60**A**	1.90 ± 0.21**AB**
	H-AS	74.30 ± 1.19**C**	7.67 ± 1.95**A**	1.29 ± 0.37**B**
	CK	75.33 ± 2.28**a**	38.77 ± 3.36**a**	8.03 ± 0.43**a**
	L-NS	58.63 ± 2.21**b**	32.90 ± 2.25**ab**	6.83 ± 0.20**ab**
C soybean	M-NS	52.17 ± 2.06**bc**	29.47 ± 1.53**bc**	6.17 ± 0.48**bc**
	H-NS	49.00 ± 2.02**c**	27.77 ± 2.03**bc**	5.40 ± 0.49**bc**
	L-AS	53.73 ± 3.42**bc**	32.57 ± 2.19**ab**	6.03 ± 0.24**bc**
	M-AS	46.57 ± 2.15**c**	25.77 ± 2.52**bc**	5.07 ± 0.49**c**
	H-AS	38.77 ± 2.91**d**	21.43 ± 3.26**c**	4.57 ± 0.83**c**

### Analysis of the ionome

**Figure 1:**
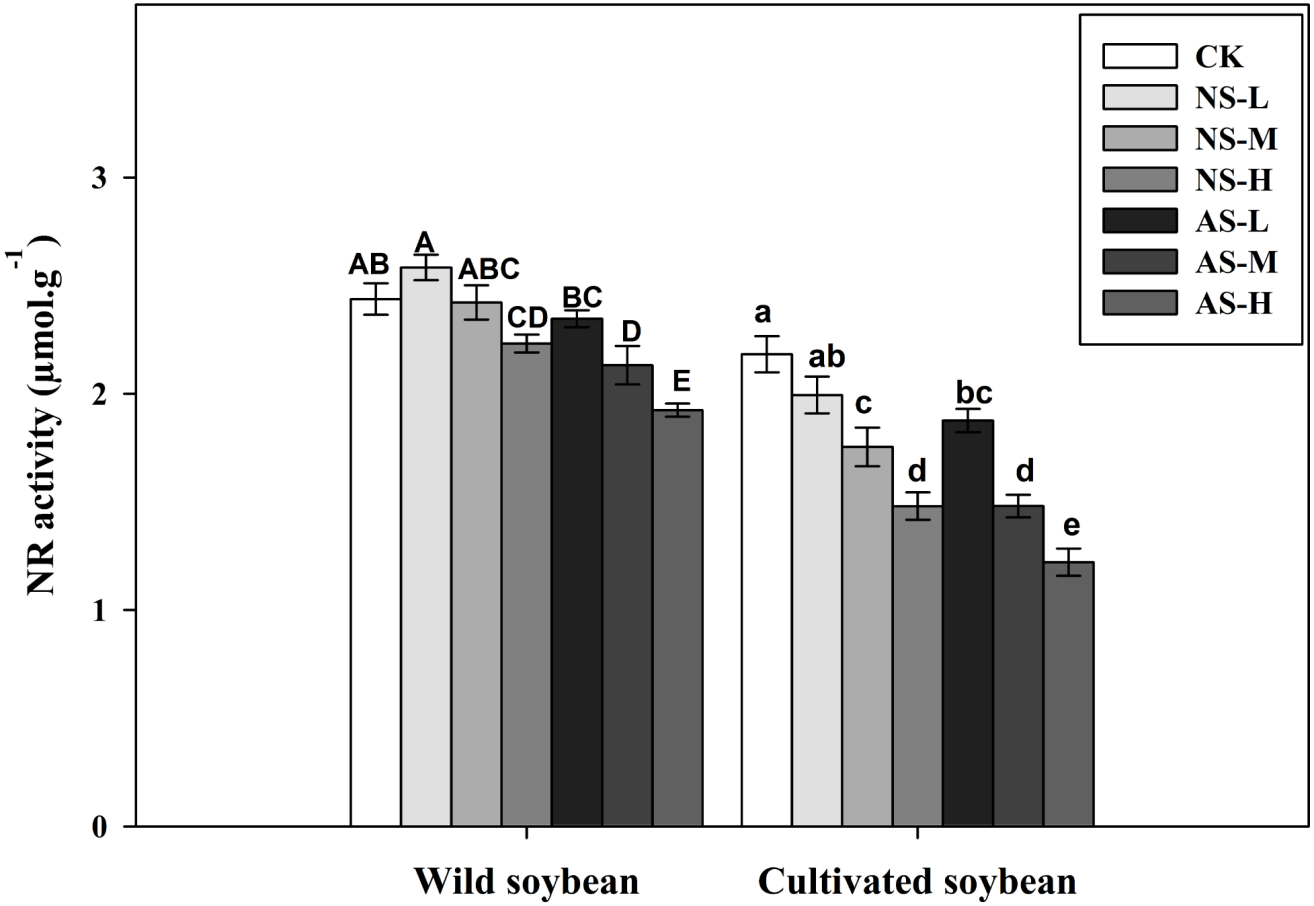
Changes in nitrate reductase (NR) activity of wild and cultivated soybean leaves under control and salt stress treatments. Bars indicate SE of mean, *n* = 3 and different letters (A–G and a–g) above the columns indicate significant differences at *P* < 0.05 according to Duncan’s method. W, wild soybean; C, cultivated soybean; CK, Control; L-NS, M-NS and H-NS represent low (10 mmol L^−1^), medium (20 mmol L^−1^) and high (30 mmol L^−1^) concentrations of saline salt stress, respectively; while L-AS, M-AS and H-AS represent low (10 mmol L^−1^), medium (20 mmol L^−1^) and high (30 mmol L^−1^) concentrations of alkaline salt stress, respectively.

PCA showed different responses to saline and alkaline salt stress with respect to the accumulation of ions by roots of the two soybean accessions (Figs. 1A–1D). The Na^+^ concentration in both soybean accessions showed trends of significant increases in response to increases in the concentrations of either stress. Wild soybean roots accumulated significantly (*P* < 0.05) more Na^+^ than did cultivated soybean roots, i.e., 238.77% relative to CK versus 196.90%, respectively, for 30 mmol L^−1^ alkaline stress and 144.00% versus 111.72%, respectively, for 30 mmol L^−1^ saline stress (Table 3).

**Table 2:** Changes in root length, root fresh weight and dry weight of wild and cultivated soybean under control and salt stress conditions. The values are shown as mean ± SE. Different letters (A-G and a-g) mark significantly different values at *P* < 0.05 according to Duncan’s method. W, wild soybean; C, cultivated soybean; CK, Control; L-NS, M-NS, and H-NS represent low (10 mmol L^−1^), medium (20 mmol L^−1^) and high (30 mmol L^−1^) concentrations of saline salt stress, respectively; while L-AS, M-AS and H-AS represent low (10 mmol L^−1^), medium (20 mmol L^−1^) and high (30 mmol L^−1^) concentrations of alkaline salt stress, respectively.

Variety	Treatment	Root length (cm)	Root fresh weight (g)	Root dry weight (g)
	CK	31.77 ± 1.01**AB**	3.90 ± 0.79**A**	0.70 ± 0.13**A**
	L-NS	32.40 ± 0.46**A**	3.80 ± 0.55**A**	0.57 ± 0.06**AB**
	M-NS	32.40 ± 1.04**A**	3.70 ± 0.21**A**	0.49 ± 0.03**AB**
W soybean	H-NS	30.77 ± 1.72**AB**	3.23 ± 0.20**A**	0.49 ± 0.08**AB**
	L-AS	29.70 ± 1.65**AB**	3.47 ± 0.42**A**	0.50 ± 0.04**AB**
	M-AS	29.13 ± 1.13**AB**	3.20 ± 0.36**A**	0.45 ± 0.06**B**
	H-AS	28.07 ± 1.32**B**	2.73 ± 0.27**A**	0.38 ± 0.04**B**
	CK	33.07 ± 1.97**a**	13.30 ± 0.61**a**	2,34 ± 0.26**a**
	L-NS	29.90 ± 1.19**ab**	12.70 ± 0.86**a**	1.74 ± 0.13**b**
	M-NS	29.37 ± 2.08**abc**	11.97 ± 0.39**a**	1.68 ± 0.25**b**
C soybean	H-NS	28.77 ± 0.93**abc**	10.23 ± 0.32**a**	1.36 ± 0.04**bc**
	L-AS	29.37 ± 1.74**abc**	12.00 ± 0.50**a**	1.45 ± 0.14**b**
	M-AS	25.47 ± 1.37**bc**	9.80 ± 3.11**a**	1,23 ± 0.07**bc**
	H-AS	24.37 ± 1.15**c**	8.87 ± 3.07**a**	0.89 ± 0.08**c**

For both stresses, Ca^2+^, K^+^, Mg^2+^ and P^3+^ concentrations in the roots of both species showed trends of significant decreases in response to increasing stress, in comparison with the control (Table 3, *P* < 0.05). A more pronounced decrease in Ca^2+^, K^+^, Mg^2+^ and P^3+^ concentrations was observed in plants subjected to alkali stress (38.20, 29.96, 26.20 and 46.48%, respectively, in wild soybean and 55.53, 51.17, 42.54 and 51.50%, respectively, in cultivated soybean under 30 mmol L^−1^ alkaline stress), than in plants subjected to saline stress (20.29, 15.45, 15.66 and 21.03%, respectively, in wild and 38.74, 35.95, 30.20 and 41.77%, respectively, in cultivated soybean under 30 mmol L^−1^ saline stress). Both salt stresses significantly increased B^3+^ concentration in the roots of the two accessions (Table 4, *P* < 0.05). The 10 and 20 mmol L^−1^ concentrations of both stresses exhibited similar increases in B^+^ concentration in both soybean accessions. A more pronounced increase was observed in plants subjected to 30 mmol L^−1^ alkaline stress (81.55% in wild soybean and 63.01% in cultivated soybean) than in plants subjected to 30 mmol L^−1^ saline stress (58.68% in wild soybean and 52.24% in cultivated soybean).

Low saline and alkaline stress concentrations (10 mmol L^−1^) significantly increased Fe^3+^ concentrations in roots of both accessions, whereas medium (20 mmol L^−1^) and high (30 mmol L^−1^) saline or alkaline concentrations significantly increased Fe^3+^ concentration in wild soybean roots but significantly reduced them in cultivated soybean roots (Table 4, *P* < 0.05). The Zn^2+^ and Mn^2+^ concentrations in the roots of both soybean accessions showed significant decreasing trends in response to increasing concentrations of either salt treatment (Table 4). The root Zn^2+^ and Mn^2+^ concentrations of both soybean accessions showed significant decreasing trends under both salt treatments (Table 4, *P* < 0.05). The concentrations of Zn^2+^ and Mn^2+^ decreased more in plants subjected to 30 mmol L^−1^ alkaline stress (28.74% and 25.86% in wild soybean, respectively, and 40.86% and 50.45% in cultivated soybean, respectively) than in plants subjected to 30 mmol L^−1^ saline stress (29.51% and 20.98% in wild soybean, respectively, and 36.38% and 32.71% in cultivated soybean, respectively). With increasing concentrations of both stresses, the root Cu^2+^ concentration increased in both soybean accessions, significantly so in the case of wild soybean, where the increase for alkali stress was significantly greater than that for saline stress (Table 4, *P* < 0.05).

**Table 3:** Changes in Na^+^, Ca^2+^, K^+^, Mg^2+^ and P^3+^ contents of wild and cultivated soybean roots under control and salt stress conditions. The values are shown as mean ± SE. Different letters (A–G and a-g) mark significantly different values at *P* < 0.05according to Duncan’s method. W, wild soybean; C, cultivated soybean; CK, Control; L-NS, M-NS, and H-NS represent low (10 mmol L^−1^), medium (20 mmol L^−1^) and high (30 mmol L^−1^) concentrations of saline salt stress, respectively; while L-AS, M-AS and H-AS represent low (10 mmol L^−1^), medium (20 mmol L^−1^) and high (30 mmol L^−1^) concentrations of alkaline salt stress, respectively.

Variety	Treatment	Na^+^	Mg^2+^	Ca^2+^	K^+^	P^3+^
	CK	09.87 ± 0.33**G**	27.65 ± 0.47**A**	41.05 ± 1.43**A**	205.84 ± 2.75**A**	35.32 ± 1.48**A**
	L-NS	12.54 ± 0.00**F**	26.85 ± 0.87**A**	37.23 ± 0.25**B**	196.85 ± 1.94**B**	31.76 ± 0.60**B**
	M-NS	17.33 ± 0.65**E**	26.35 ± 0.82**A**	34.97 ± 0.27**BC**	187.60 ± 2.24**C**	28.95 ± 0.12**C**
W soybean	H-NS	24.08 ± 0.77**C**	23.32 ± 0.42**BC**	32.72 ± 0.68**C**	174.04 ± 1.24**D**	27.38 ± 0.48**C**
	L-AS	18.94 ± 0.42**D**	23.65 ± 0.94**B**	34.36 ± 0.59**C**	185.50 ± 1.98**C**	27.90 ± 0.31**C**
	M-AS	27.46 ± 0.56**B**	21.45 ± 0.58**CD**	28.22 ± 0.76**D**	155.77 ± 2.48**E**	21.12 ± 0.34**D**
	H-AS	33.43 ± 0.52**A**	20.41 ± 0.25**D**	25.37 ± 1.06**E**	144.17 ± 2.94**F**	18.91 ± 0.91**D**
	CK	08.57 ± 0.31**e**	26.91 ± 1.27**a**	44.36 ± 0.59**a**	194.90 ± 0.77**a**	30.38 ± 0.06**a**
	L-NS	10.22 ± 0.79d**e**	23.26 ± 0.26**b**	39.55 ± 0.84**b**	169.73 ± 1.53**b**	24.56 ± 2.04**b**
	M-NS	11.77 ± 0.48**d**	20.22 ± 0.30**cd**	30.14 ± 1.32**c**	160.09 ± 1.32**c**	20.43 ± 0.26**c**
C soybean	H-NS	18.15 ± 0.56**c**	18.78 ± 0.28**d**	27.18 ± 1.00**d**	124.83 ± 2.59**e**	17.69 ± 0.27**d**
	L-AS	16.06 ± 0.69**c**	21.78 ± 0.28**bc**	37.27 ± 0.71**b**	152.19 ± 2.08**d**	23.62 ± 0.05**b**
	M-AS	21.08 ± 1.34**b**	16.68 ± 0.07**e**	23.75 ± 1.53**e**	117.78 ± 1.51**e**	18.01 ± 0.30**cd**
	H-AS	25.46 ± 0.56**a**	15.46 ± 0.84**e**	19.73 ± 0.08**f**	95.16 ± 4.90**f**	14.73 ± 0.68**e**

**Table 4:** Changes in Mn^2+^, B^3+^, Fe^3+^, Cu^2+^ and Zn^2+^ contents of wild and cultivated soybean roots under control and salt stress conditions. The values are shown as mean ± SE. Different letters (A-G and a-g) mark significantly different values at *P* < 0.05 according to Duncan’s method. W, wild soybean; C, cultivated soybean; CK, Control; L-NS, M-NS, and H-NS represent low (10 mmol L^−1^), medium (20 mmol L^−1^) and high (30 mmol L^−1^) concentrations of saline salt stress, respectively; while L-AS, M-AS and H-AS represent low (10 mmol L^−1^), medium (20 mmol L^−1^) and high (30 mmol L^−1^) concentrations of alkaline salt stress, respectively.

Variety	Treatment	Mn^2+^	B^3+^	Fe^3+^	Cu^2+^	Zn^2+^
	CK	0.12 ± 0.01**A**	0.34 ± 0.01**E**	0.06 ± 0.00**C**	0.02 ± 0.00**C**	0.12 ± 0.00**A**
	L-NS	0.11 ± 0.00**AB**	0.37 ± 0.00**C**	0.13 ± 0.00**AB**	0.02 ± 0.00**AB**	0.10 ± 0.00A**B**
	M-NS	0.10 ± 0.01**AB**	0.46 ± 0.01**C**	0.09 ± 0.00**BC**	0.02 ± 0.00**BC**	0.09 ± 0.01**BC**
W soybean	H-NS	0.09 ± 0.00**AB**	0.54 ± 0.02**C**	0.08 ± 0.12**BC**	0.02 ± 0.00**C**	0.08 ± 0.01**BC**
	L-AS	0.10 ± 0.01**AB**	0.39 ± 0.01**B**	0.16 ± 0.05**A**	0.03 ± 0.00**A**	0.10 ± 0.01**C**
	M-AS	0.10 ± 0.01**AB**	0.57 ± 0.01**AB**	0.10 ± 0.01**ABC**	0.02 ± 0.00**AB**	0.09 ± 0.00**BC**
	H-AS	0.09 ± 0.01**B**	0.61 ± 0.02**A**	0.08 ± 0.00**BC**	0.02 ± 0.00**C**	0.08 ± 0.00**C**
	CK	0.12 ± 0.00**a**	0.28 ± 0.01**d**	0.06 ± 0.01**bc**	0.02 ± 0.00**a**	0.11 ± 0.01**a**
	L-NS	0.11 ± 0.01**b**	0.32 ± 0.01**cd**	0.09 ± 0.00**ab**	0.02 ± 0.00**a**	0.09 ± 0.00**b**
	M-NS	0.09 ± 0.00**c**	0.39 ± 0.01**b**	0.05 ± 0.00**c**	0.02 ± 0.00**a**	0.09 ± 0.00**bc**
C soybean	H-NS	0.08 ± 0.00**cd**	0.43 ± 0.03**ab**	0.05 ± 0.00**c**	0.02 ± 0.00**a**	0.07 ± 0.00**de**
	L-AS	0.09 ± 0.00**c**	0.33 ± 0.01**c**	0.12 ± 0.03**a**	0.02 ± 0.00**a**	0.08 ± 0.00**cd**
	M-AS	0.08 ± 0.01**d**	0.41 ± 0.01**b**	0.05 ± 0.00**bc**	0.02 ± 0.00**a**	0.07 ± 0.00**de**
	H-AS	0.06 ± 0.00**e**	0.46 ± 0.01**a**	0.04 ± 0.01**c**	0.02 ± 0.00**a**	0.06 ± 0.00**e**

With respect to changes in anion concentration in response to salt stress, the Cl^−^ concentration increased significantly in salt-treated roots of both soybean accessions, with the increase in wild soybean roots being clearly greater (*P* < 0.05) than in cultivated soybean roots under the same level of saline stress (Table 4, *P* < 0.05). Similarly, the NO_3_^−^ concentration in roots of both soybean accessions and the SO_4_^2−^ concentration in cultivated soybean roots showed significant decreasing trends with increasing concentrations of the two stresses, with a more pronounced impact being observed under alkaline than under saline stress (Table 4, *P* < 0.05). Moreover, H_2_PO_4_^−^ concentration decreased in response to increasing stress in roots of cultivated soybean grown under both stresses and in wild soybean under alkaline stress. The SO_4_^2−^ and H_2_PO_4_^−^ concentrations of wild soybean roots showed significant increasing trends under saline stress. Moreover, in wild soybean, the increase in SO_4_^2−^ concentration (5.95, 10.62 and 30.31%) was greater than that of H_2_PO_4_^−^ (0.78, 6.81 and 13.03%, relative to that of the control) under 10, 20 and 30 mmol L^−1^ saline stress, respectively. When subjected to alkaline stress, SO_4_^2−^ concentration in wild soybean increased at low alkaline stress (10 mmol L^−1^) by 1.47% whereas it decreased by 0.47% and 10.25% at medium and high alkaline stress (10 and 20 mmol L^−1^), respectively (Table 4).

**Table 5:** Changes in NO_3_^−^, SO_4_^2−^, Cl^−^, H_2_PO_4_^−^, C_2_O_4_^2−^ contents of wild and cultivated soybean roots under control and salt stress conditions. The values are shown as mean ± SE. Different letters (A-G and a-g) mark significantly different values at *P* < 0.05 according to Duncan’s method. W, wild soybean; C, cultivated soybean; CK, Control; L-NS, M-NS, and H-NS represent low (10 mmol L^−1^), medium (20 mmol L^−1^) and high (30 mmol L^−1^) concentrations of saline salt stress, respectively; while L-AS, M-AS and H-AS represent low (10 mmol L ^−1^), medium (20 mmol L^−1^) and high (30 mmol L^−1^) concentrations of alkaline salt stress, respectively.

Variety	Treatment	NO_3_^−^	SO_4_^2−^	Cl^−^	H_2_PO_4_^−^	C_2_O_4_^2−^
	CK	8.52 ± 0.97**A**	21.19 ± 0.60**C**	4.18 ± 0.06**F**	2.81 ± 0.07**BC**	1.07 ± 0.02**D**
	L-NS	8.16 ± 0.39**A**	22.45 ± 0.32**BC**	6.95 ± 0.51**C**	2.84 ± 0.00BC	1.17 ± 0.03**D**
	M-NS	7.66 ± 0.18**AB**	23.44 ± 0.13**B**	9.16 ± 0.21**B**	3.00 ± 0.11**AB**	1.21 ± 0.00**CD**
W soybean	H-NS	6.70 ± 0.06**BC**	25.49 ± 0.73**A**	10.34 ± 0.18**A**	3.18 ± 0.06**A**	1.18 ± 0.09**D**
	L-AS	7.51 ± 0.40**AB**	21.50 ± 0.06**C**	5.30 ± 0.03**E**	2.62 ± 0.13**CD**	1.41 ± 0.07**C**
	M-AS	6.62 ± 0.09**BC**	21.09 ± 0.62**C**	6.05 ± 0.12**D**	2.50 ± 0.12**DE**	1.63 ± 0.11**B**
	H-AS	5.56 ± 0.11**C**	19.02 ± 0.39**D**	6.81 ± 0.09**C**	2.30 ± 0.02**F**	1.94 ± 0.07**A**
	CK	7.27 ± 0.17**a**	22.91 ± 0.85**a**	5.55 ± 0.19**d**	2.94 ± 0.00**a**	1.35 ± 0.018**b**
	L-NS	6.46 ± 0.31**b**	21.91 ± 0.85**ab**	6.69 ± 0.04**bc**	2.80 ± 0.04**ab**	1.38 ± 0.20**b**
	M-NS	5.99 ± 0.02**b**	20.20 ± 1.50**abc**	7.23 ± 0.36**ab**	2.68 ± 0.15**bc**	1.42 ± 0.11**b**
C soybean	H-NS	5.15 ± 0.35**c**	18.36 ± 1.24**c**	7.93 ± 0.40**a**	2.58 ± 0.03**c**	1.42 ± 0.05**b**
	L-AS	5.88 ± 0.23**b**	21.70 ± 3.00**ab**	6.03 ± 0.41**cd**	2.49 ± 0.05**c**	1.54 ± 0.06**ab**
	M-AS	4.52 ± 0.26**c**	18.86 ± 0.74**bc**	6.27 ± 0.01**cd**	2.05 ± 0.02**d**	1.73 ± 0.00**ab**
	H-AS	3.47 ± 0.09**d**	17.30 ± 2.18**c**	6.80 ± 0.06**bc**	1.85 ± 0.03**e**	1.83 ± 0.11**a**

Additionally, the C_2_O_4_^2−^ concentration accumulated significantly in roots of both soybean accessions exposed to salt stress, with a greater increase in plants subjected to alkaline stress than to saline stress. The wild soybean accession accumulated significantly more C_2_O_4_^2−^ than did the cultivated soybean accession under all concentrations of both stresses,with a more pronounced increase in plants subjected to 30 mmol L^−1^ alkaline stress (80.85% in wild soybean and 36.23% in cultivated soybean) than in plants subjected to 30 mmol L^−1^ saline stress (9.66% in wild soybean and 5.26% in cultivated soybean) (Table 5) (Dataset S1 and Dataset S2).

**Figure 2:**
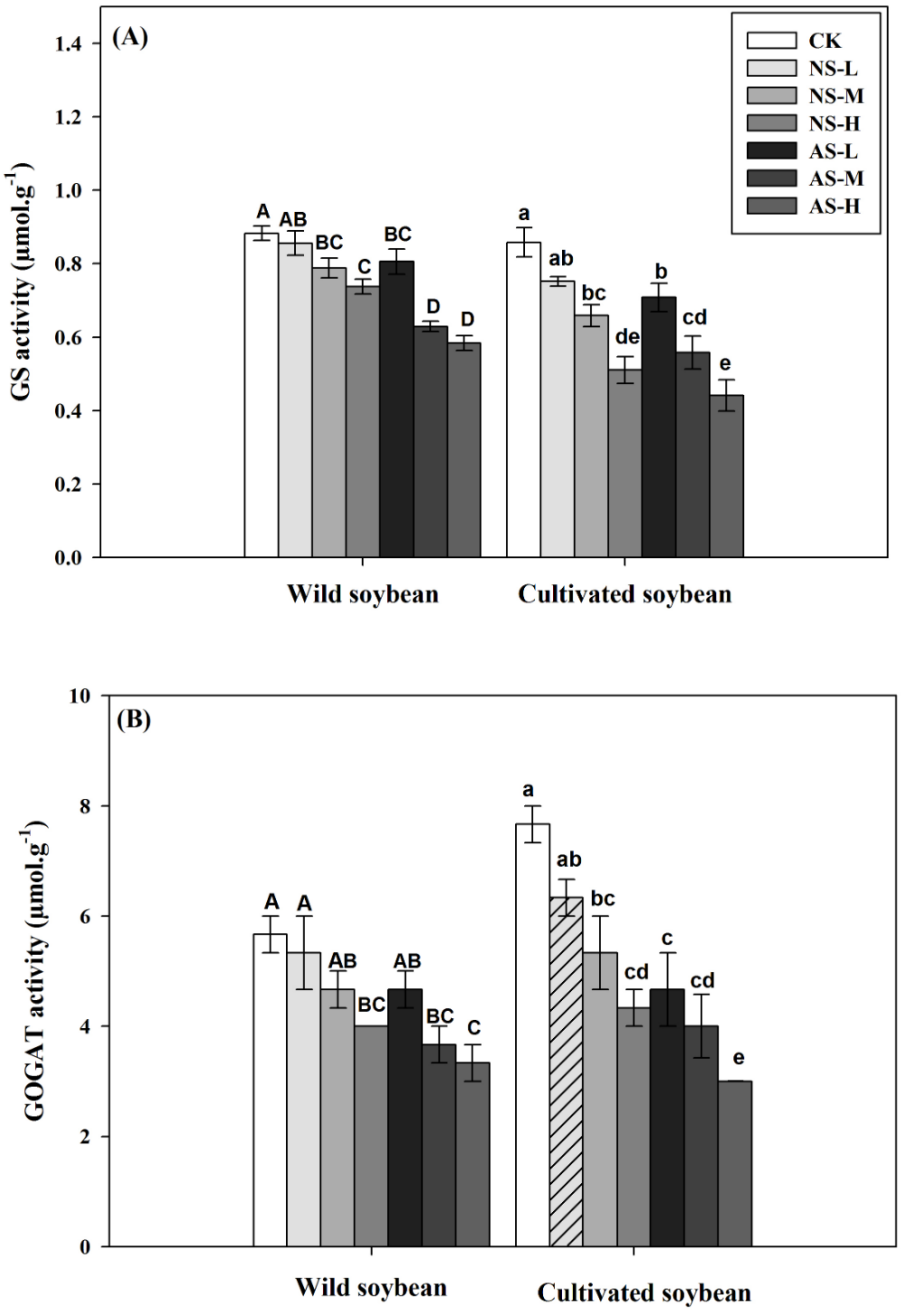
Changes in GS (A) and GOGAT (B) activities of wild and cultivated soybean leaves under control and salt stress treatments. Bars indicate SE of mean, *n* = 3 and different letters (A–G and a–g) above the columns indicate significant differences at *P* < 0.05 according to Duncan’s method. W, wild soybean; C, cultivated soybean; CK, Control; L-NS, M-NS and H-NS represent low (10 mmol L^−1^), medium (20 mmol L^−1^) and high (30 mmol L^−1^) concentrations of saline salt stress, respectively; while L-AS, M-AS and H-AS represent low (10 mmol L ^−1^), medium (20 mmol L^−1^) and high (30 mmol L^−1^) concentrations of alkaline salt stress, respectively.

### Changes in photosynthesis parameters

PCA showed an obvious distinction between the photosynthetic capacities of the two soybean species in response to increasing salt and alkali concentrations (Figs. 2A–2D). Concentrations of photosynthetic pigments (Chla, Chlb, and Chl t) of both accessions showed significantly decreasing trends in response to increasing salt stress (Table 6, *P* < 0.05). The decrease was significantly higher in cultivated soybean than wild soybean, particularly in plants subjected to alkaline stress. Moreover, significant decreasing trends were also observed in the Car concentrations of cultivated and wild soybean under both salt and alkaline stress, respectively (Table 4, *P* < 0.05).The Car concentration in wild soybean did not increase significantly under saline stress. Similarly, photosynthetic gaseous exchange parameters of the two soybean accessions responded differently to the two salt stresses. The *P*_n_, *G*_s_, *C*_i_/ *C*_a_ and *E* in cultivated soybean showed significant decreasing trends in response to increasing salt stress (Table 7, *P* < 0.05), while water-use efficiency (*WUE*) did not decrease significantly under both stresses, regardless of the level of stress (Table 7). The wild soybean accession showed significantly higher *P*_*n*_ and *G*_s_ at 10, 20 and 30 mmol L^−1^saline stress, higher *WUE* at 10, 20 and 30 mmol L ^−1^saline stress and higher *E* at 10 mmol L ^−1^saline stress, relative to that of the control (Table 7, *P* < 0.05) (Dataset S1 and Dataset S2).

### Changes in nitrogen assimilation enzyme activities

**Figure 3:**
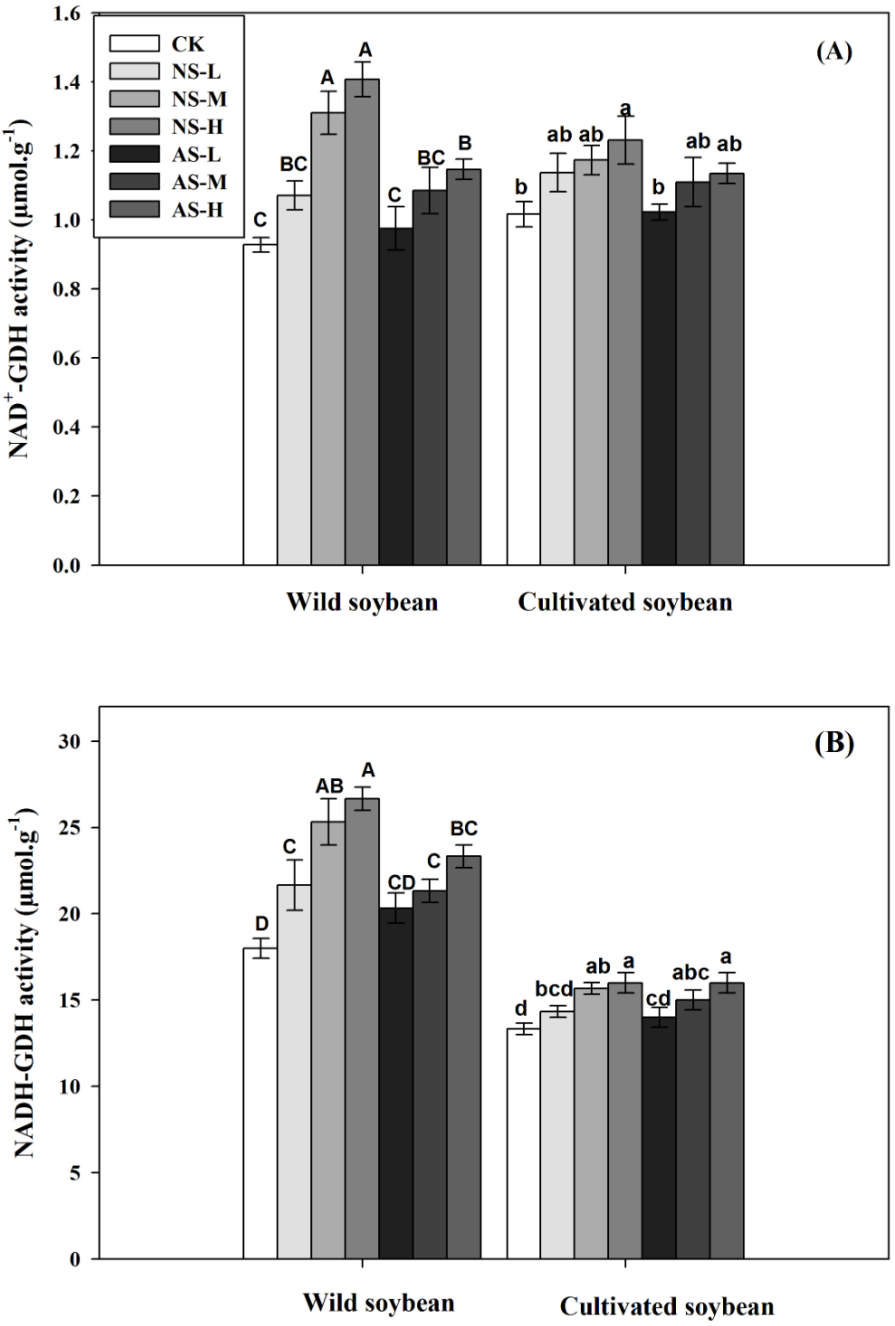
Changes in NAD-GDH (A) and NADH-GDH (B) activities of wild and cultivated soybean leave under control and salt stress treatments. Bars indicate SE of mean, *n* = 3 and different letters (A–G and a–g) above the columns indicate significant differences at *P* < 0.05 according to Duncan’s method. W, wild soybean; C, cultivated soybean; CK, Control; L-NS, M-NS and H-NS represent low (10 mmol L^−1^), medium (20 mmol L^−1^) and high (30 mmol L^−1^) concentrations of saline salt stress, respectively; while L-AS, M-AS and H-AS represent low (10 mmol L^−1^), medium (20 mmol L^−1^) and high (30 mmol L^−1^) concentrations of alkaline salt stress, respectively.

**Figure 4:**
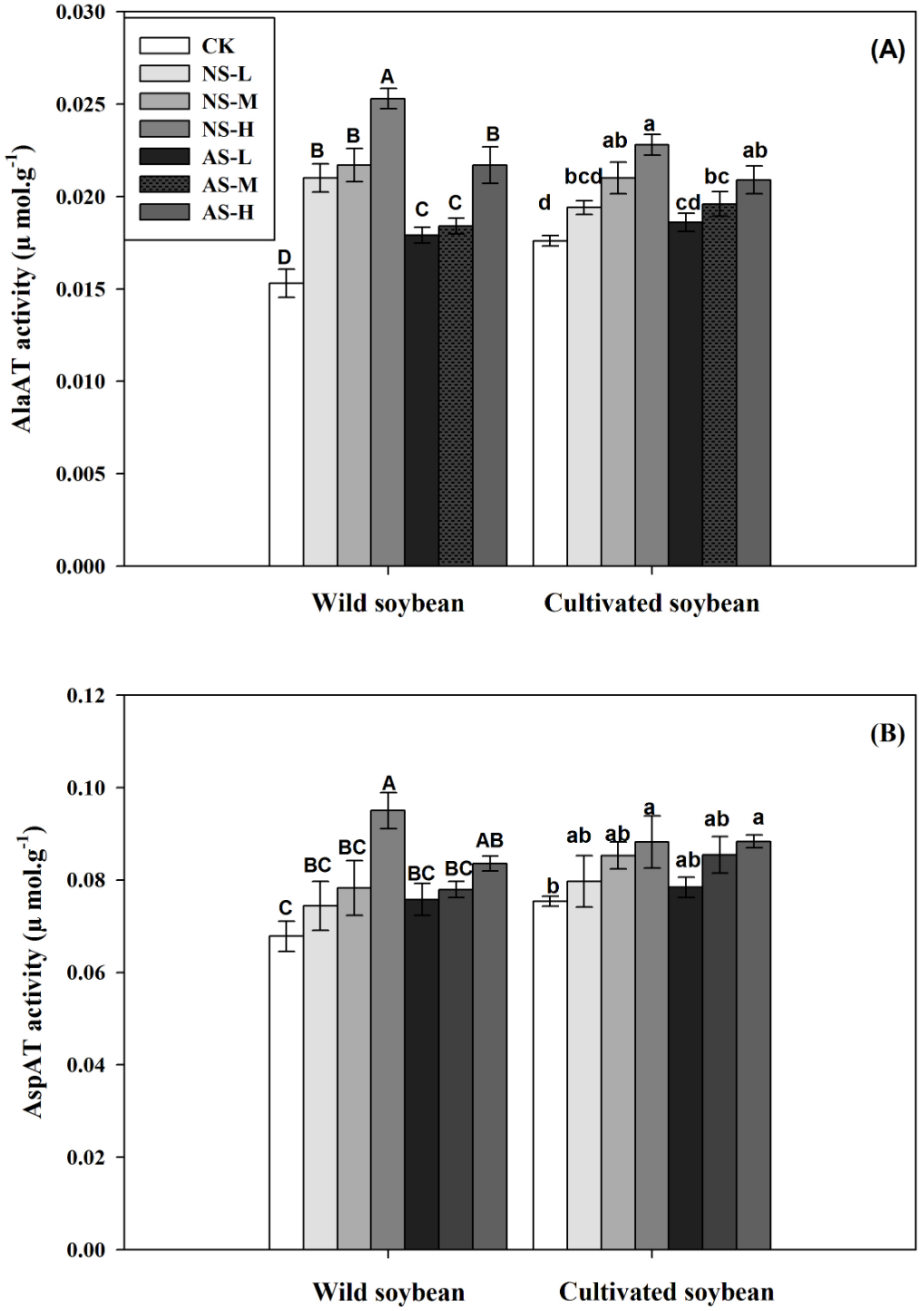
Changes in AlaAT (A) and AspAT (B) activities of wild and cultivated soybean leaves under control and salt stress treatments. Bars indicate SE of mean, *n* = 3 and different letters (A–G and a–g) above the columns indicate significant differences at *P* < 0.05 according to Duncan’s method. W, wild soybean; C, cultivated soybean; CK, Control; L-NS, M-NS and H-NS represent low (10 mmol L^−1^), medium (20 mmol L^−1^) and high (30 mmol L^−1^) concentrations of saline salt stress, respectively; while L-AS, M-AS and H-AS represent low (10 mmol L^−1^), medium (20 mmol L^−1^) and high (30 mmol L^−1^) concentrations of alkaline salt stress, respectively.

PCA and Loading plot results (Figs. 3A–3D) showed different responses of nitrogen assimilation enzyme activities exhibited by roots of the two soybean species exposed to saline/alkaline stresses. In comparison with the control, nitrate reductase (NR) activity in leaves of the cultivated soybean accession decreased significantly in response to stress by 14.02, 32.14 and 44.02% in plants subjected to 10, 20 and 30 mmol L^−1^ alkaline stress, respectively) and by 8.62, 19.61 and 32.15% in plants subjected to 10, 20 and 30 mmol L^−1^ saline stress, respectively (Fig. 4, *P* < 0.05). In the wild accession NR activity increased by 6.02% at 10 mmol L-^1^saline stress but decreased at 20 and 30 mmol L^−1^ saline stress by 0.62% and 8.40%. The NR activity in the wild soybean also decreased in response to 10, 20 and 30 mmol L^−1^ alkaline stress, by 3.74%, 12.51% and 21.06%, respectively, compared with the unstressed control (Fig. 4).

**Table 6:** Changes in photosynthetic pigments of wild sand cultivated soybean leaves under control and salt stress conditions. The values are shown as mean ± SE. Different letters (A-G and a-g) mark significantly different values at *P* < 0.05 according to Duncan’s method. W, wild soybean; C, cultivated soybean; CK, Control; L-NS, M-NS, and H-NS represent low (10 mmol L^−1^), medium (20 mmol L^−1^) and high (30 mmol L^−1^) concentrations of saline salt stress, respectively; while L-AS, M-AS and H-AS represent low (10 mmol L^−1^), medium (20 mmol L^−1^) and high (30 mmol L^−1^) concentrations of alkaline salt stress, respectively.

Variety	Treatment	Chl a (mg g-1 DW)	Chl b (mg g-1 DW)	Chla+b (mg g-1 DW)	Car (mg g-1 DW)
	CK	15.78 ± 0.24**A**	5.06 ± 0.12**A**	21.81 ± 0.64**A**	3.31 ± 0.07**A**
	L-NS	15.69 ± 0.36**AB**	4.83 ± 0.25A	20.01 ± 0.24**B**	3.41 ± 0.21**A**
	M-NS	15.07 ± 0.27**ABC**	4.56 ± 0.33**A**	18.88 ± 0.38**BC**	3.50 ± 0.72**A**
W soybean	H-NS	14.52 ± 0.23**C**	4.43 ± 0.24**A**	17.90 ± 0.52**C**	3.84 ± 0.65**A**
	L-AS	14.79 ± 0.48**BC**	4.33 ± 0.47**A**	18.18 ± 0.63**C**	2.98 ± 0.33**AB**
	M-AS	12.84 ± 0.14**D**	2.86 ± 0.22**B**	15.86 ± 0.61**D**	2.93 ± 0.35**AB**
	H-AS	12.73 ± 0.10**D**	2.42 ± 0.22**B**	15.17 ± 0.23**D**	1.77 ± 0.21**C**
	CK	16.50 ± 0.24**a**	6.04 ± 0.43**a**	21.54 ± 0.54**a**	3.20 ± 0.16**a**
	L-NS	15.86 ± 0.32**a**	4.32 ± 0.25**b**	20.19 ± 0.48**b**	2.84 ± 0.06**ab**
	M-NS	14.17 ± 0.37**c**	3.81 ± 0.12**bc**	17.98 ± 0.49**c**	2.41 ± 0.44**abc**
C soybean	H-NS	13.69 ± 0.10**c**	3.38 ± 0.31**bcd**	17.07 ± 0.26**c**	2.56 ± 0.02**abc**
	L-AS	14.90 ± 0.22**b**	3.40 ± 0.19**bcd**	18.29 ± 0.36**c**	2.04 ± 0.28**bcd**
	M-AS	11.56 ± 0.05**d**	3.02 ± 0.48**cd**	14.58 ± 0.43**d**	1.70 ± 0.49**cd**
	H-AS	10.52 ± 0.10**e**	2.44 ± 0.26**d**	12.96 ± 0.37**e**	1.16 ± 0.21**d**

**Table 7:** Changes in gas exchange parameter of wild and soybean leaves under control and salt stress conditions. The values are shown as mean ± SE. Different letters (A–G and a–g) mark significantly different values at *P* < 0.05 according to Duncan’s method. W, wild soybean; C, cultivated soybean; CK, Control; L-NS, M-NS, and H-NS represent low (10 mmol L^−1^), medium (20 mmol L^−1^) and high (30 mmol L^−1^) concentrations of saline salt stress, respectively; while L-AS, M-AS and H-AS represent low (10 mmol L^−1^), medium (20 mmol L^−1^) and high (30 mmol L^−1^) concentrations of alkaline salt stress, respectively.

Variety	Treatment	*Pn* (CO_2_m^−2^ s^−1^)	*Gs* (mol m^−2^ s^−1^)	*E* (µmol H_2_O m^−2^ s^−1^)	*WUE* (*P*_*n*_*/E*)	*Ci/Ca* (cm^3^m^−3^)
	CK	12.57 ± 0.24**B**	1670.33 ± 8.95**C**	10.33 ± 0.15**B**	1.22 ± 0.04**BC**	0.97 ± 0.00**A**
	L-NS	12.70 ± 0.12**B**	1680.67 ± 10.59**C**	11.13 ± 0.12**A**	1.14 ± 0.01**CD**	0.91 ± 0.00**B**
	M-NS	12.73 ± 0.26**B**	1767.00 ± 28.00**B**	9.93 ± 0.09**B**	1.28 ± 0.01**B**	0.87 ± 0.00**C**
W soybean	H-NS	13.80 ± 0.10**A**	1891.33 ± 6.64**A**	9.10 ± 0.15**C**	1.52 ± 0.03**A**	0.83 ± 0.01**D**
	L-AS	11.43 ± 0.03**C**	1477.67 ± 8.09**D**	9.03 ± 0.48**C**	1.27 ± 0.07**B**	0.86 ± 0.01**C**
	M-AS	9.43 ± 0.20**D**	1398.00 ± 40.38**E**	8.43 ± 0.12**C**	1.12 ± 0.03**CD**	0.83 ± 0.01**D**
	H-AS	8.27 ± 0.34**E**	1386.67 ± 5.70**E**	7.57 ± 0.27**D**	1.09 ± 0.03**D**	0.78 ± 0.01**E**
	CK	11.07 ± 0.03**a**	1167.33 ± 21.67**ab**	9.43 ± 0.12**a**	1.17 ± 0.02**a**	0.93 ± 0.00**a**
	L-NS	10.53 ± 0.03**a**	1199.67 ± 39.83**a**	9.23 ± 0.09**a**	1.14 ± 0.01a	0.89 ± 0.03**b**
	M-NS	9.67 ± 0.07**b**	1151.67 ± 23.47**ab**	8.53 ± 0.15**ab**	1.13 ± 0.01**a**	0.79 ± 0.00**d**
C soybean	H-NS	6.83 ± 0.03**d**	793.33 ± 3.28**bc**	7.07 ± 0.09**b**	0.97 ± 0.02**a**	0.68 ± 0.00**f**
	L-AS	8.43 ± 0.9**c**	1132.33 ± 7.88**ab**	8.07 ± 0.09**ab**	1.05 ± 0.00**a**	0.84 ± 0.00**c**
	M-AS	7.93 ± 0.29**c**	871.67 ± 105.73a**bc**	7.30 ± 1.30**b**	1.18 ± 0.26**a**	0.75 ± 0.01**e**
	H-AS	5.13 ± 0.42**e**	609.67 ± 29.18**c**	5.50 ± 0.17**c**	0.94 ± 0.09**a**	0.63 ± 0.00**g**

**Figure 5:**
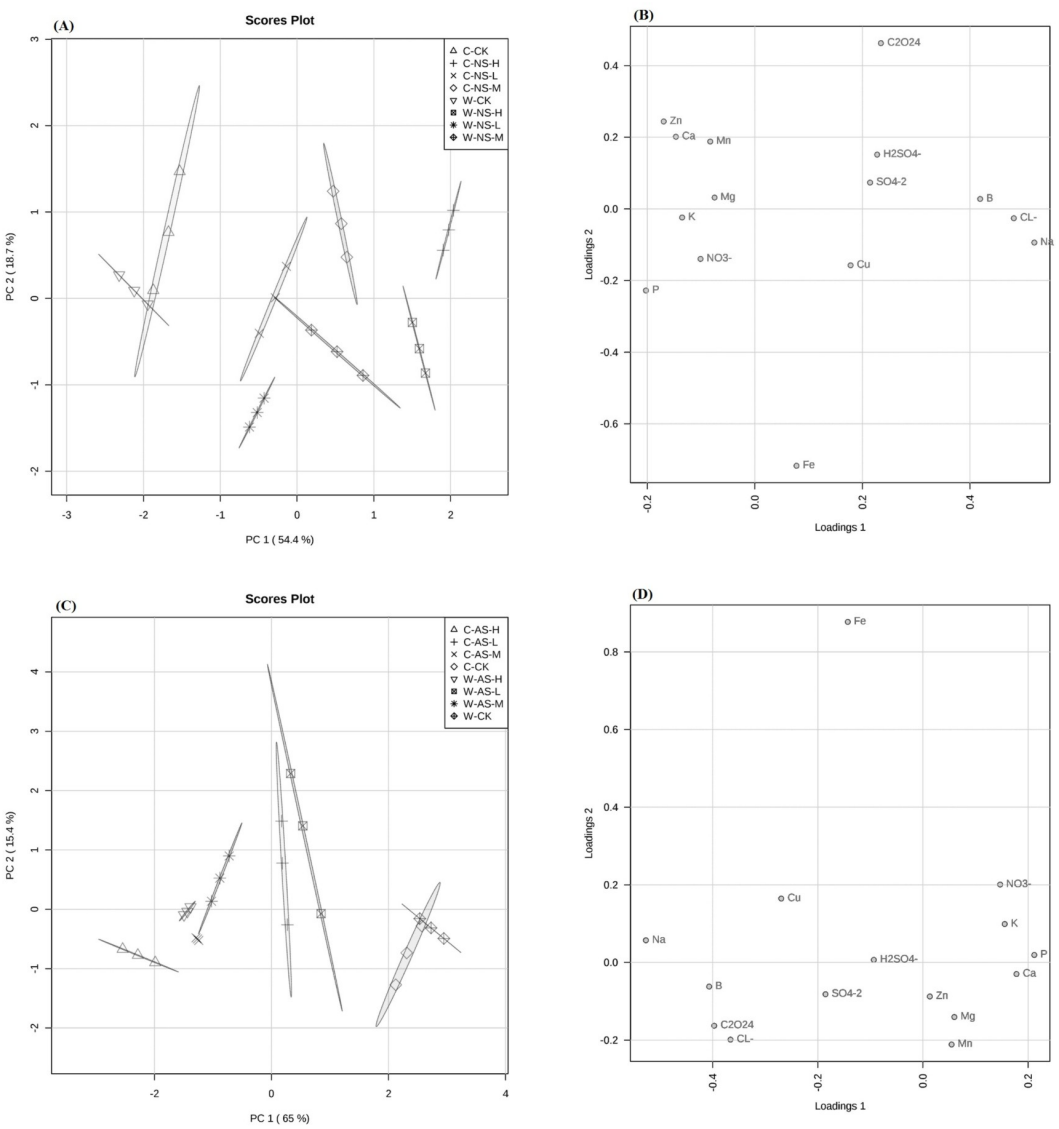
Principal component analysis (PCA) of wild and cultivated soybean seedlings ionome variation. (A) Root ionome variation among neutral salt stress samples. (B) Loadings of ions to PC1 and PC2 in roots among neutral salt stress samples (C) Root ionome variation among alkaline salt stress samples. (D) loadings of ions to PC1 and PC2 in roots among alkaline salt stress samples.

**Figure 6:**
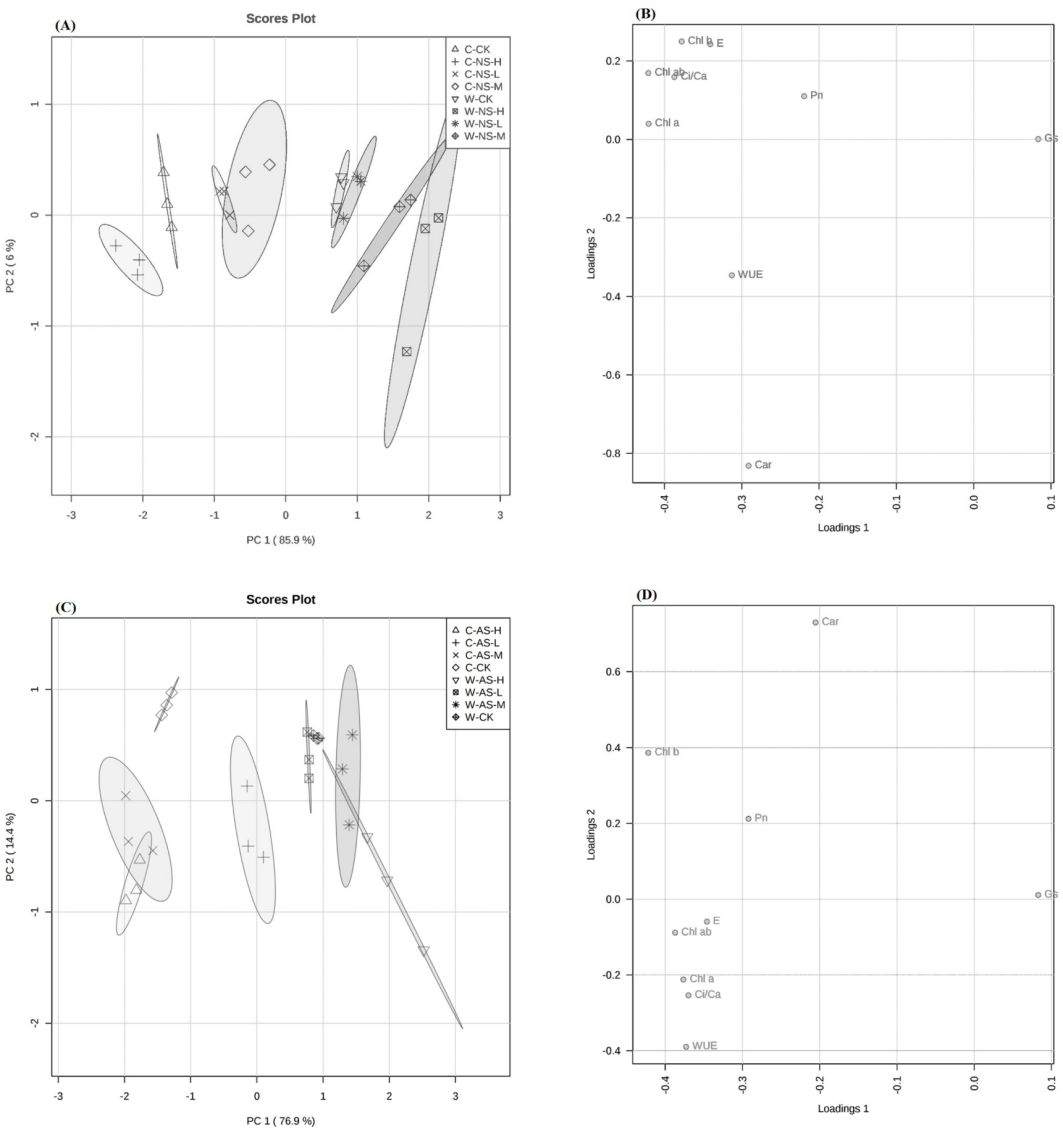
Principal component analysis (PCA) of wild and cultivated soybean seedlings photosynthetic parameters. (A) Leves photosynthetic parameters among neutral salt samples. (B) Loadings of Photosynthetic parameters to PC1 and PC2 in roots among neutral salt stress samples. (C) Leves photosynthetic parameters among alkaline salt stress samples. (D) Loadings of Photosynthetic parameters to PC1 and PC2 in roots among alkaline salt stress samples.

**Figure 7:**
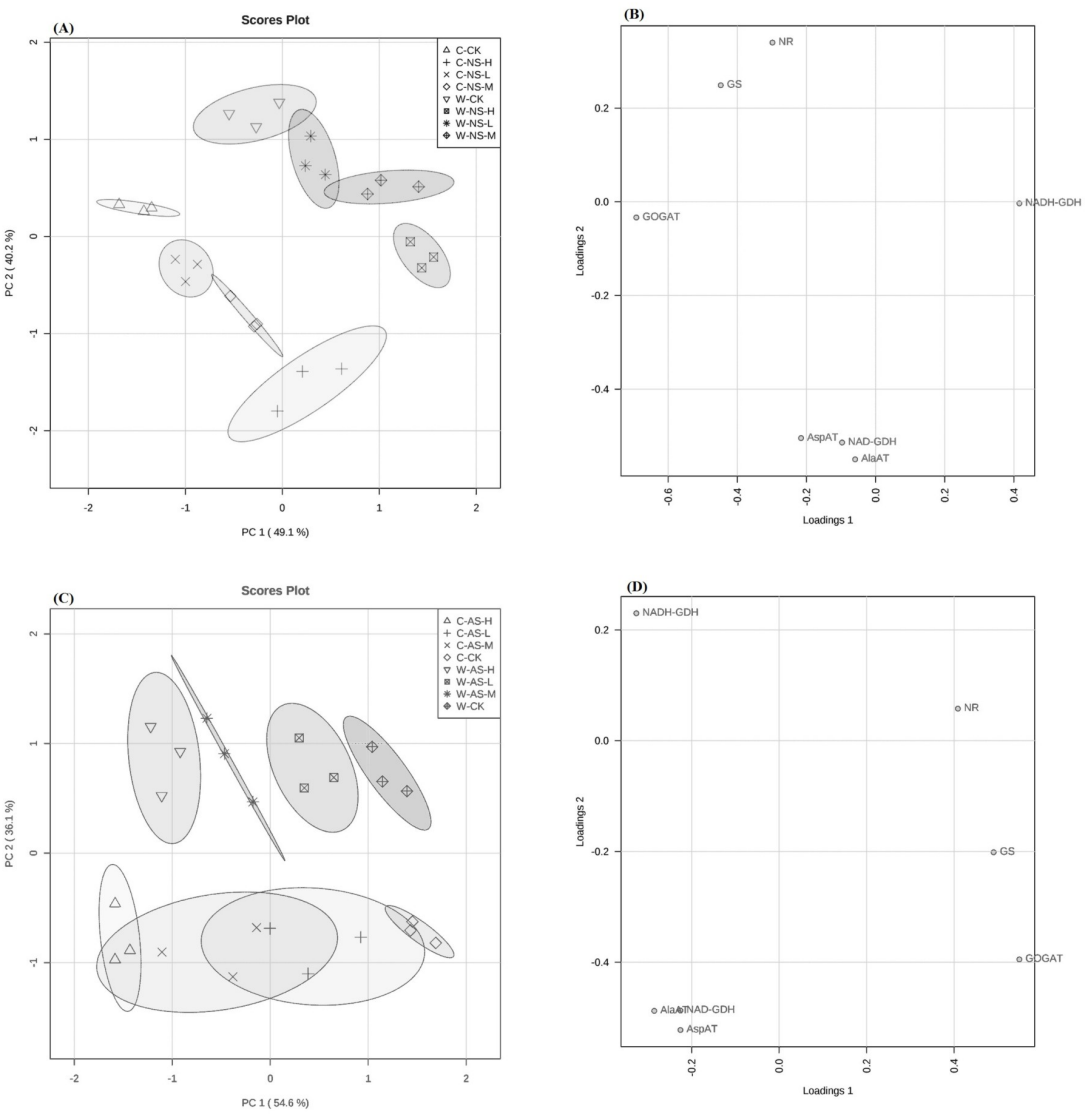
Principal component analysis (PCA) of nitrogen metabolizing enzymes in wild and cultivated soybean seedlings under saline and alkaline salt stresses. (A) Leaves nitrogen metabolizing enzymes among neutral salt stress samples. (B) Loadings of nitrogen metabolizing enzymes to PC1 and PC2 among neutral salt stress samples. (C) Leaves nitrogen metabolizing enzymes among alkaline salt stress samples (D) Loadings of nitrogen metabolizing enzymes to PC1 and PC2 among alkaline salt stress samples.

In response to increasing concentrations of either salt treatment, glutamine synthetase/glutamine synthase (GS/GOGAT ) activities decreased significantly in leaves of both accessions (Figs. 5A, 5B; *P* < 0.05). The decrease was clearly greater in the cultivated soybean than in the wild soybean. The decrease in GS and GOGAT activities was more pronounced in plants subjected to 30 mmol L^−1^ alkali stress (33.96% and 41.18%, respectively, in the wild soybean and 48.84% and 60.87%, respectively, in the cultivated soybean) than in the corresponding plants subjected to saline stress (16.22% and 29.41%, respectively, in the wild accession and 40.70% and 43.48%, respectively, in the cultivated accession) (Figs. 5A, 5B). In response to increases in salt concentrations of both stresses, the activities of glutamate dehydrogenase (GDH), alanine aminotransferase (AlaAT) and aspartate aminotransferase (AspAT) all increased significantly in leaves of both soybean accessions, with the increase in wild soybean leaves being higher than that in cultivated soybean (Figs. 6A, 6B, Figs. 7A, 7B; *P* < 0.05).The increases in glutamate dehydrogenase (NAD-GDH, NADH-GDH), AlaAT and AspAT activities were more pronounced in plants subjected to 30 mmol L^−1^ saline stress (51.70, 48.15, 40.12 and 65.36%, respectively), in wild soybean relative to the control plants, than in plants subjected to alkaline stress (23.56, 29.63, 23.30 and 41.83%, respectively). Similarly, the increases in NAD-GDH, NADH-GDH, AlaAT and AspAT activities in cultivated soybean were more pronounced in plants subjected to 30 mmol L^−1^ saline stress (21.14, 20.00, 16.98 and 29.55%, respectively), relative to the control plants) than in alkaline stress (11.66, 20.00, 17.11 and 18.75%, respectively) (Figs. 6A, 6B, 7A, 7B; *P* < 0.05) (Dataset S1 and Dataset S2).

## Discussion

Soil salinity is a major abiotic stress factor that significantly reduces growth and yield of economically important crop plants. In the present study, the growth performance of salt-treated seedlings of both cultivated and wild soybean accessions decreased significantly under both saline and alkaline salt stresses. The response of plants to alkaline stress was different from that in response to saline salt stress. Alkaline stress usually causes fructan inhibition, accumulation of organic acids (Guo et al., 2010), root membrane disorganization, depletion of root oxygen, mineral nutrient deficiency as well as ion imbalance between root cells and the surrounding environment. Such salt-mediated effects seriously reduce plant growth and metabolism (Yang et al., 2007). The adaptation of seedlings to alkaline stress also needs more nutrients and energy than does adaptation to saline stress (Jiao et al., 2018). In the present study, growth inhibition in plants subjected to alkali salt stress was more pronounced than that of plants subjected to saline stress, probably due to the high pH induced by alkaline stress (Wang & Li, 2011).

The wild soybean accession showed greater photosynthetic adaptation to saline and alkaline stresses by retaining more photosynthetic pigments than did the cultivated soybean as in consistent with previous study (Shi et al., 2015). Moreover, the salt-treated wild soybean accession exhibited higher Car concentration, which is considered to be an adaptive evolutionary trait of plants to cope with salt stress, as Car is an auxiliary pigment that can dissipate excess light energy (Duan et al., 2012).

Wild soybean showed high *E* values at 10 mmol L^−1^ saline stress, high *P*_*n*_ and *G*_s_, at 10, 20 and 30 10 mmol L^−1^ saline stress and high *WUE* at 20 and 30 mmol L^−1^ saline stress (relative to that of the control) as supported by our previous study (Shao et al., 2016; Jiao et al., 2018). Such photosynthetic adaptations under high salinity levels are frequently observed in halophytes in which photosynthesis remains normal or even increases under salt stress (Rabhi et al., 2012). Thus, wild soybean seems to be able to adjust the photosynthetic machinery to adapt to saline stress. It should be noted that the wild soybean accession in the current study also had a significantly higher photosynthetic rate than did the cultivated accession under normal conditions, which might have been a beneficial trait for stress acclimation.

On the other hand, the levels of *E*, *G*_s_, and *C*_i_/*C*_a_ decreased in cultivated soybean in response to increasing concentrations of both stresses and in wild soybean under increasing concentrations of alkaline stress. Additionally, the decrease in Chlt and Car concentrations was higher in cultivated soybean than in wild soybean. These findings indicate that the decrease in photosynthetic rate was limited by both photosynthetic and stomatal factors in the cultivated accession, but by only stomatal factors in the wild soybean genotype (Zheng, Xiao & Jin, 2013). The decrease in *WUE* was clearly greater for cultivated soybean than for wild soybean. The salt tolerance of a plant is associated with high water-use efficiency (Paridaa & Dasa, 2005). Thus the increased *WUE* in wild soybean at 10 and 20 mmol L^−1^ saline stress might have been beneficial with respect to stress acclimation.

The root system plays a key role in plant salt tolerance through a series of adaptative mechanisms in response to various adverse conditions (Yang et al., 2017). Na^+^ and Cl^−^ are extremely toxic to plants (Teakle & Tyerman, 2010). They accumulated in the roots of both soybean accessions, which might have been beneficial to stress acclimation by reducing their transport to aerial plant parts and hence protecting leaves from the salt damage, leaving the important process of photosynthesis unaffected. Their accumulation was higher in the roots of the wild soybean accession than in the cultivated soybean. Thus, wild soybean seems to better able to regulate ions to adapt to salt stress (Jiao et al., 2018). Plants usually avoid excessive Na^+^ build-up by accumulating anions (Yang et al., 2007; Munns & Tester, 2008) rather than by synthesizing organic osmoprotectant compounds, as the synthesis of organic compounds is expensive in terms of materials and energy (Munns, 2002).

High C_2_O_4_^2−^ concentration accumulated in the roots of both soybean species in response to stress, with greater accumulation occurring in plants subjected to alkaline stress, and this might have been one of the reasons for the marked decrease in growth of both soybean accessions (Yang et al., 2007). The wild soybean accumulated C_2_O_4_^2−^ markedly under alkaline stress as consistent to previous study (Yang et al., 2007) as well as showing better maintenance of inorganic ions under both stresses. These might have been adaptative strategies beneficial to stress acclimation by minimizing and alleviating ion toxicity (Jiao et al., 2018). For both stresses, the NO_3_^−^ and Cl^−^ content in roots of both soybean accessions showed antagonistic interactions as consistent with previous studies (Abdelgadir, Oka & Fujiyama, 2005). The decrease in NO_3_^−^ uptake might have decreased nitrogen assimilation and the synthesis of amino acids and proteins, resulting in a greater reduction in dry weight of both soybean accessions, relative to that of the control (Queiroz, Sodek & Haddad, 2012). Moreover, both salt stresses greatly reduced the activities of enzymes of leaf nitrogen assimilation in both soybean species. The nitrate reductase (NR) activity in leaves of cultivated soybean decreased with increasing concentrations of both stresses, but only under alkaline stress for the wild soybean, with the corresponding reductions being greater in cultivated than in wild soybean. Thus, decreased NR activity seems to be the result of decreased NO_3_^−^ absorption rather than the direct effect of salt stresses (Abdelgadir, Oka & Fujiyama, 2005; Meng et al., 2016). Our findings support those reported from other studies (Bybordi & Ebrahimian, 2011; Shao et al., 2015; Meng et al., 2016).

The glutamine synthetase/glutamine synthase (GS/GOGAT) cycle links plant nitrogen and carbon assimilation by binding inorganic nitrogen (NH_4_^+^) to the carbon skeleton, a step necessary for plant growth and development (Forde & Lea, 2007). In response to either stress, GS/GOGAT activities decreased significantly in leaves of both soybean accessions, which indicates their inability to incorporate NH_4_^+^ properly, to fill the glutamate pool necessary for proline biosynthesis (Wang et al., 2007). Previous studies had shown that the toxic Na^+^ and Cl^−^ accumulation in rice plants subjected to salt stress damaged the chloroplasts and photorespiration, resulting in decreased GS/GOGAT activities (Meng et al., 2016).

GS/GOGAT activities were higher in leaves of the wild soybean accession following exposure to stress.Thus, the wild soybean seems to have synthesized more nitrogen-containing compounds than has the cultivated soybean, an adaptation which might have been beneficial to stress acclimation (Lancien, Gadal & Hodges, 2000). The salt-induced reductionin the GS/GOGAT cycle was previously reported for different crop plants (Sahu, Sahoo & Sahoo, 2001; Wang et al., 2007; Shao et al., 2015; Meng et al., 2016). Moreover, the high aminating glutamate dehydrogenase (NADH-GDH) activity in leaves of both soybean species has accompanied the reduced GS/GOGAT cycle by incorporating NH_4_^+^ directly onto glutamate (Wang et al., 2007; Wang et al., 2014). Glutamate is a key intermediate of nitrogen metabolism and a substrate for the synthesis of amino acids, nucleotides, glutathione and chlorophyll (Forde & Lea, 2007; Martinez-Andujar et al., 2013). The affinity of NADH-GDH for NH_4_^+^ is usually very low, compared with the GS enzyme (Wang et al., 2007), so that the increased NADH-GDH activity in stressed plants reflects the presence of high concentrations of NH_4_^+^ in both soybean accessions following stress exposure. Moreover, it can also be concluded that the GS/GOGAT cycle might have been inhibited due to the damaging effect of salt stress rather than to substrate limitation. The accumulation of NH_4_^+^ ions reflects reduced nitrogen assimilation and decreased plant growth (Wang et al., 2007).The salt-induced increase in NADH-GDH activity was previously reported from different crop plants (Kwinta & Cal, 2004; Wang et al., 2007; Gao et al., 2013; Naliwajski & Skłodowska, 2018).

The deamination of glutamate to 2-oxoglutarate and NH_4_^+^ ions is very important under stress conditions (Bechtold, Pahlich & Lea, 1998). The high deaminating glutamate dehydrogenase (NAD-GDH) activity in both soybean accessions might be a source of 2-oxoglutarate and NH_4_^+^ ions. The high NAD-GDH activity testified to its roles in the catabolism of carbohydrates, through the supply of 2-oxoglutarate for the Krebs cycle as well as that of reduction force in the shape of NAD(P)H, necessary for many metabolic processes (El-Shora & Abo-Kassem, 2001; Kwinta & Cal, 2004; Liu & Von Wirén, 2017). The increased NAD-GDH activity might play an important role in replenishing the reduced carbon pool in both soybean accessions (Naliwajski & Skłodowska, 2018). Both aminating and deaminating activities of GDH were higher in leaves of wild soybean, relative to that of the cultivated soybean, on exposure to stress. Thus, wild soybean seems to have developed a strong compensatory mechanism of delivering carbon sources and incorporating NH_4_^+^ to avoid a shortage of amino acids necessary for osmotic adjustments of the cell.

High alanine aminotransferase (AlaAT) activity in both soybean accessions reflects the disturbed Krebs cycle and activation of the reversible reaction of pyruvate and glutamate to alanine and 2-oxoglutarate.Therefore, this effect seems to have facilitated ATP production Van Dongen et al., (2011). The transamination of aspartate to oxaloacetate by the aspertate aminotransferase (AspAT) enzyme is an important physiological anaplerotic reaction of the Krebs cycle (Van Dongen et al.,, 2011). The increased activities of AlaAT, AspAT, and NADH-GDH enzymes in both soybean accessions might have been attributed to the high glutamate demand and maintenance of Krebs cycle to achieve correction of the carbon:nitrogen ratio under stress conditions (Forde & Lea, 2007; Naliwajski & Skłodowska, 2018). Increased AlaAT and AspAT activities under salt stress have previously been reported for different crop plants (Surabhi et al., 2008; Gao et al., 2013; Naliwajski & Skłodowska, 2018).

## Conclusions

Both saline and alkaline stresses had negative impact on the growth and metabolism of the wild and cultivated soybean species, as indicated by decreased growth and photosynthetic parameters, ion regulation and decreased activity of enzymes involved in nitrogen assimilation, with greater decreases in plants subjected to alkaline stress than in plants subjected to saline stress. The aminating glutamate dehydrogenase (NADH-GDH) played a complementary role in NH_4_^+^ assimilation of both soybean accession in response to stress. Moreover, increased activities of alanine aminotransferase (AlaAT), aspertate aminotransferase (AspAT), and NADH-GDH in stressed plants reflect the high glutamate demand and maintenance of the Krebs cycle for the correction of the carbon: nitrogen ratio in both soybean accessions. The activities of these enzymes were higher in wild soybean than in cultivated soybean in response to increasing concentrations of both stresses. In the presence of salt stress, the wild soybean better regulated the physiological mechanism of photosynthesis and nitrate assimilation than did the cultivated soybean. The present study provides a theoretical basis to screen and utilize wild soybeans for the breeding of new stress-tolerant soybean varieties.

##  Supplemental Information

10.7717/peerj.8191/supp-1 Data S1 Raw data

10.7717/peerj.8191/supp-2 Data S2 Analyzed data

Abbreviations

AlaAT: alanine aminotransferase
AspAT: aspartate aminotransferase
Car: carotenoids
Chl a: chlorophyll a
Chl b: chlorophyll b
Chl t: total chlorophyll
***C***_***1***_∕***C***_***a***_: ratio of sub-stomatal to atmospheric CO_2_ concentration
*E*: transpiration rate
GDH: glutamate dehydrogenase
***G***_***s***_: stomatal conductance
GS/GOGAT: glutamine synthetase/glutamine synthase
NAD-GDH: deaminating glutamate dehydrogenase
NADH-GDH: aminating glutamate dehydrogenase
NR: nitrate reductase
***P***_***n***_: net CO_2_ assimilation rate
PCA: principal component analysis
TCA: tricarboxylic acid
WUE: Water-use efficiency

## Additional Information and Declarations

### Data Availability

The following information was supplied regarding data availability: The raw data are provided in the Supplementary Files.
